# Clinical evaluation of the three-dimensional printed strut-type prosthesis combined with autograft reconstruction for giant cell tumor of the distal femur

**DOI:** 10.3389/fonc.2023.1206765

**Published:** 2023-08-22

**Authors:** Linyun Tan, Ye Li, Xin Hu, Minxun Lu, Yuqi Zhang, Yuxiong Gan, Chongqi Tu, Li Min

**Affiliations:** ^1^ Department of Orthopedic Surgery and Orthopedic Research Institute, West China Hospital, Sichuan University, Chengdu, China; ^2^ Department of Model Worker and Innovative Craftsman, West China Hospital, Sichuan University, Chengdu, China; ^3^ Department of Orthopedics, West China Hospital, Sichuan University/West China School of Nursing, Sichuan University, Chengdu, China; ^4^ Key Lab for Biomechanical Engineering of Sichuan Province, Sichuan University, Chengdu, China

**Keywords:** giant cell tumor of bone, 3D-printed prosthesis, bone cement, subchondral bone, porous titanium

## Abstract

**Propose:**

This study aimed to describe the design and surgical techniques of a three-dimensional (3D) printed strut-type prosthesis with a porous titanium surface for distal femur giant cell tumors of bone (GCTB) and evaluate the short-term clinical outcomes.

**Methods:**

From June 2018 to January 2021, 9 consecutive patients with grade I or II GCTB in the distal femur underwent extended intralesional curettage followed by 3D-printed strut-type prosthesis combined with autograft reconstruction were retrospectively reviewed to assess their clinical and radiographic outcomes.

**Results:**

All patients were followed up for 30.8 ± 7.5 months (18–42 months) after surgery. The mean affected subchondral bone percentage and the mean subchondral bone thickness before surgery was 31.8% ± 9.6% (range, 18.2% ~50.2%) and 2.2 ± 0.8 mm (range, 1.2-4.0 mm), respectively. At the final follow-up, all the patients were alive without local recurrence; no postoperative complications were observed. Patients had significant improvements in postoperative MSTS-93 score [(26.7 ± 2.4) vs. (18.8 ± 3.7), *P <* 0.05], and ROM [(122.8° ± 9.1°) vs. (108.3° ± 6.1°), *P <* 0.05] compared with their preoperative statuses. Furthermore, the mean subchondral bone thickness has increased to 10.9 ± 1.3 mm (range, 9.1-12.1 mm).

**Conclusion:**

3D-printed strut-type prosthesis combined with autograft reconstruction provides acceptable early functional and radiographic outcomes in patients with grade I or II GCTB in distal femur due to the advantages of the prosthesis such as good biocompatibility, osseointegration capacity, and subchondral bone protection. If our early outcomes can be further validated in studies with more patients and sufficient follow-up, this method may be evaluated as an alternative for the treatment of grade I or II GCTB in the distal femur.

## 1 Introduction

Giant cell tumor of bone (GCTB) is a locally aggressive benign tumor, which accounts for about 5% of all primary bone tumors (1). It commonly affects young adults aged between 20 and 45 years, with the distal femur being the most common site of occurrence (2). Based on the radiograph appearance, GCTB can be classified into three grades (I-III) according to the Campanacci grading system (3). For grades I or II GCTBs in the distal femur, curettage combined with adjuvant therapies is the mainstream surgical treatment aimed at completely removing the tumor while preserving knee function as much as possible (4, 5). In the currently available literature, there is currently no consensus regarding cementation’s influence on knee joint degeneration, but some studies have reported that the use of bone cement may increase the risk of mechanical failure for GCTB patients following extended curettage. For example, XU et al. (6) found that secondary degenerative changes occurred in 30.3% (23/76) of the patients with GCT around the knee who were treated with extensive curettage and cementation. A similar finding has been reported in other studies, the prevalence of osteoarthritis after curettage and application of PMMA ranges from 4% to 25% in extremities (7–10). Moreover, extended curettage itself and intra-articular pathological fractures have been mentioned as potential risk factors for the emergence of secondary osteoarthritis (11–13).

As an effective shock absorber, the integrity of the subchondral bone is essential for the knee joint function. It plays important role in maintaining the function of the knee joint, while a lower quantity of subchondral bone may lead to degenerative changes, deformity of the articular surface, and cartilage damage. Numerous studies have reported that enough subchondral bone remaining layer could decrease the possibilities of postoperative degenerative changes and mechanical failure of the knee joint (14–16). For example, Abdelrahman et al. (14) stated that patients whose residual thickness of the knee subchondral bone was less than 10 mm has a 2.5-fold higher risk of joint degeneration compared to those whose residual thickness was more than 10 mm. Therefore, how to achieve the effective reconstruction of the cavity bone defect with the articular subchondral bone being protected is of great importance for patients’ long-term prognoses.

Since Baddeley et al. (17) first reported the application of cement packing in the treatment of GCTB around the knee in the 1970s, bone cement has gradually become the most popular reconstruction material for the cavity bone defects following curettage of GCTBs of the extremities due to its advantages of easier use, complete voids filling, sufficient mechanical strength, and ease of detection of recurrence. Moreover, bone cement has been claimed to have a tumoricidal ability by its exothermic reaction (18). Nevertheless, this exothermic reaction is regarded as a double-edged sword possessing both tumoricidal and subchondral bone-damaging effects. The application of bone cement immediately adjacent to the subchondral bone following extended intralesional curettage has been suggested to be at an increased risk of thermal damage to the subchondral bone. Given this, some have suggested that the subchondral bone grafting (≥1 cm) is used as an allograft buffer to prevent thermal necrosis. Numerous studies have confirmed that this “sandwich technique”, extended curettage + subchondral bone grafting + cement packing, can reduce the risk of osteoarthritis and prevent mechanical failure, which has currently been accepted as the standard reconstruction method (16, 19). However, there are still inherent problems with the bone cement filling material which can form insurmountable barriers to the aim of achieving a biological and integrated reconstruction. As bone cement is at the mercy of its poor osteoconductivity and osteoinductivity, it is impossible to achieve osseointegration and bone ingrowth on the graft-cement interface (16). Moreover, the elastic modulus for bone cement has been reported as 3.3 Gpa (20). The values for cortical bone and trabecular bone are in the ranges of 15-19 Gpa and 1.5-11.2 Gpa, respectively (21–24). This substantial difference in the elasticity modulus between the cement and the host/graft bone may result in mechanical damage and peri-cement bone resorption (9, 25). Recent studies have reported the use of calcium phosphate cement (CPC) as an alternative to PMMA in these cases. CPC demonstrates certain advantageous properties, such as bioactivity, and it presents a lower risk of exothermic reactions (26). However, there are challenges associated with the use of CPC, specifically its mechanical strength and longer setting time, which may limit its wider application (27, 28). So, further exploration and investigation are crucial to optimize therapeutic strategies for the reconstruction following curettage of GCTBs in the distal femur.

The porous titanium-based customized prosthesis has been widely developed to provide a practical solution to the above problems by using 3D printing technology. The porous titanium scaffolds are regarded as a leading replacement for bone grafts and bone cement (29, 30). They have many advantageous properties, including excellent mechanical strength, corrosion resistance, biocompatibility, and osseointegration capability (31). The porous titanium-based customized prosthesis can offer good bone ingrowth and a matchable modulus of the natural bone, while it avoids the thermal damage of cementation (32). Hence, we used a 3D-printed strut-type prosthesis combined with subchondral bone grafting to repair the cavity bone defects following extended curettage of GCTBs in the distal femur. The present study described this novel design of prosthesis and surgical technique and assessed the clinical outcomes aimed at the identification of better operative strategies.

## 2 Methods

### 2.1 Patient demographics

From June 2018 to January 2021, 9 consecutive patients with grade I or II GCTB in the distal femur, where subchondral bone remained uninvolved and the joint surface could be preserved, underwent extended intralesional curettage followed by the three-dimensional printed strut-type prosthesis combined with autograft reconstruction and were retrospectively reviewed. Patients with incomplete follow-up information, severe osteoporosis, deformities of the lower extremities, pathological fracture, and grade III GCTB or pulmonary metastasis were excluded.

**Figure 1:**
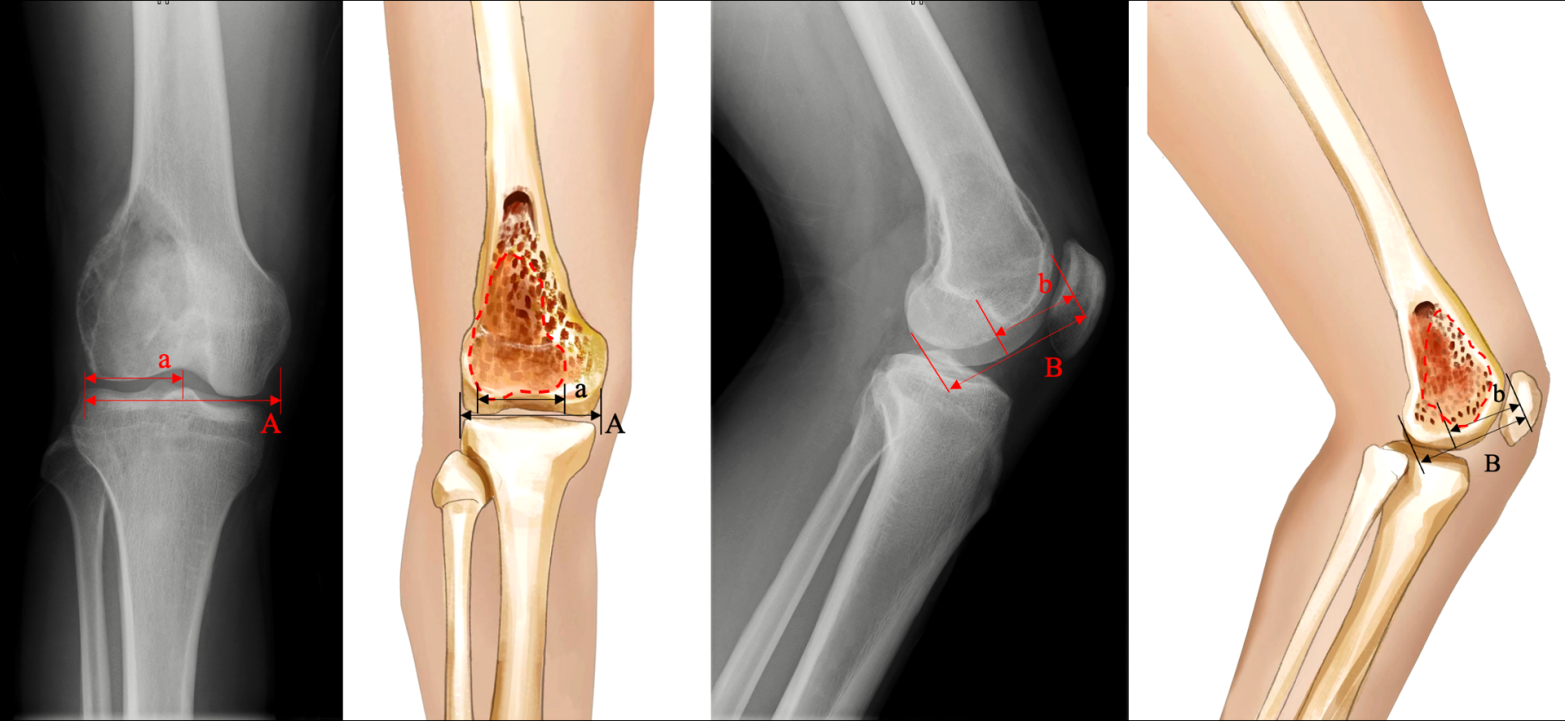
Schematic depiction of the calculation method: A= length of total subchondral bone on anteroposterior view; a= length of affected subchondral bone on anteroposterior view; B= length of total subchondral bone on lateral view; b= length of affected subchondral bone on the lateral view. The affected subchondral bone area of the distal femur was expressed as a percentage and was calculated as [a×b/(A×B)] × 100%.

The study cohort consisted of 4 males and 5 females, with a mean age of 30.8 ± 6.1 years (range, 24-44). All cases were confirmed as GCTB by punch biopsy or excision biopsy before surgery. All patients underwent a complete preoperative imaging examination, including knee X-ray, femoral 3D-CT, knee MRI, and computed tomography (SPECT); the thin-layer chest CT scan was completed for detecting possible metastatic spread to the lungs. The pre-operative assessment mainly included the following two aspects:

1) Evaluation of knee joint functions: Functional status was evaluated by the 1993 version of the Musculoskeletal Tumor Society (MSTS-93) score, which is a limb-specific assessment based on six categories (pain, function, emotional acceptance, supports, walking ability, and gait) specific to the entire lower limb (33). Each category is scored from 0 to 5, with a total score from 0 to 30 (a higher score is desirable). Additionally, the knee range of motion (ROM) was also assessed.
2) Evaluation of the subchondral bone integrity: According to the definition and method provided by Chen et al. (15), the subchondral bone was defined as affected when the distance to the tumor was less than 3 mm, while the shortest distance between the articular surface and the tumor on plain knee radiographs was defined as the residual subchondral bone thickness. Furthermore, based on the anteroposterior and lateral knee radiographs, the area of affected subchondral bone was calculated as the ratio of the affected length to the total length of the subchondral bone (**Figure 1**). In addition, Tomosynthesis Shimadzu Metal Artefact Reduction Technology (T-SMAR) can provide high-resolution cross-sectional images. By overserving those continuous images from sagittal, coronal, and transverse views, the incorporations of the morcellated autograft and the host bone can be evaluated.

**Figure 2:**
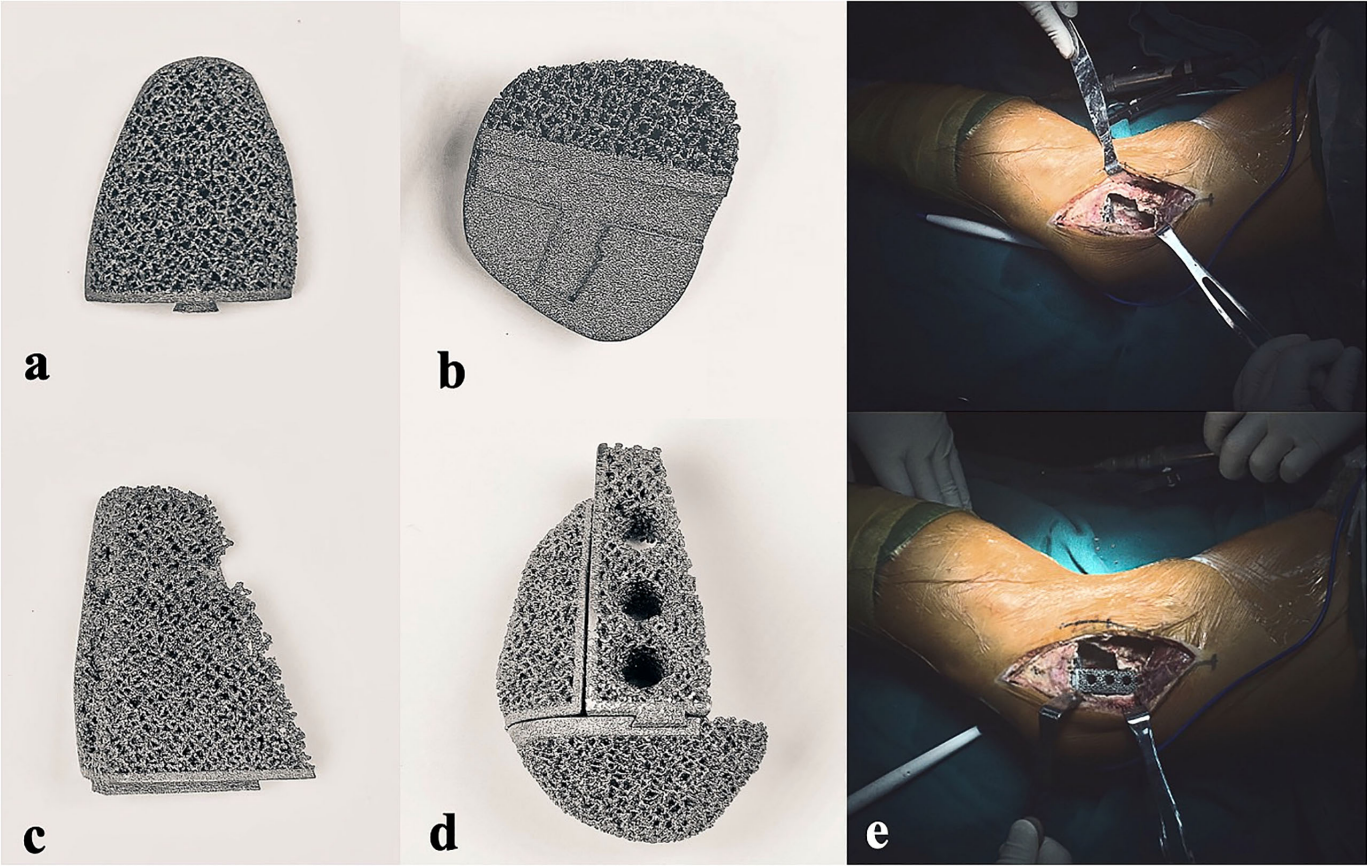
Photographs of the 3D-printed strut-type prosthesis: **(A)** The turtle shell-shaped strut **(A, B)** The turtle shell-shaped strut **(B, C)** The trapezoid-shaped strut. **(D)** The assembled prosthesis with porous titanium surface. **(E)** Intraoperative pictures of prosthesis implantation.

**Figure 3:**
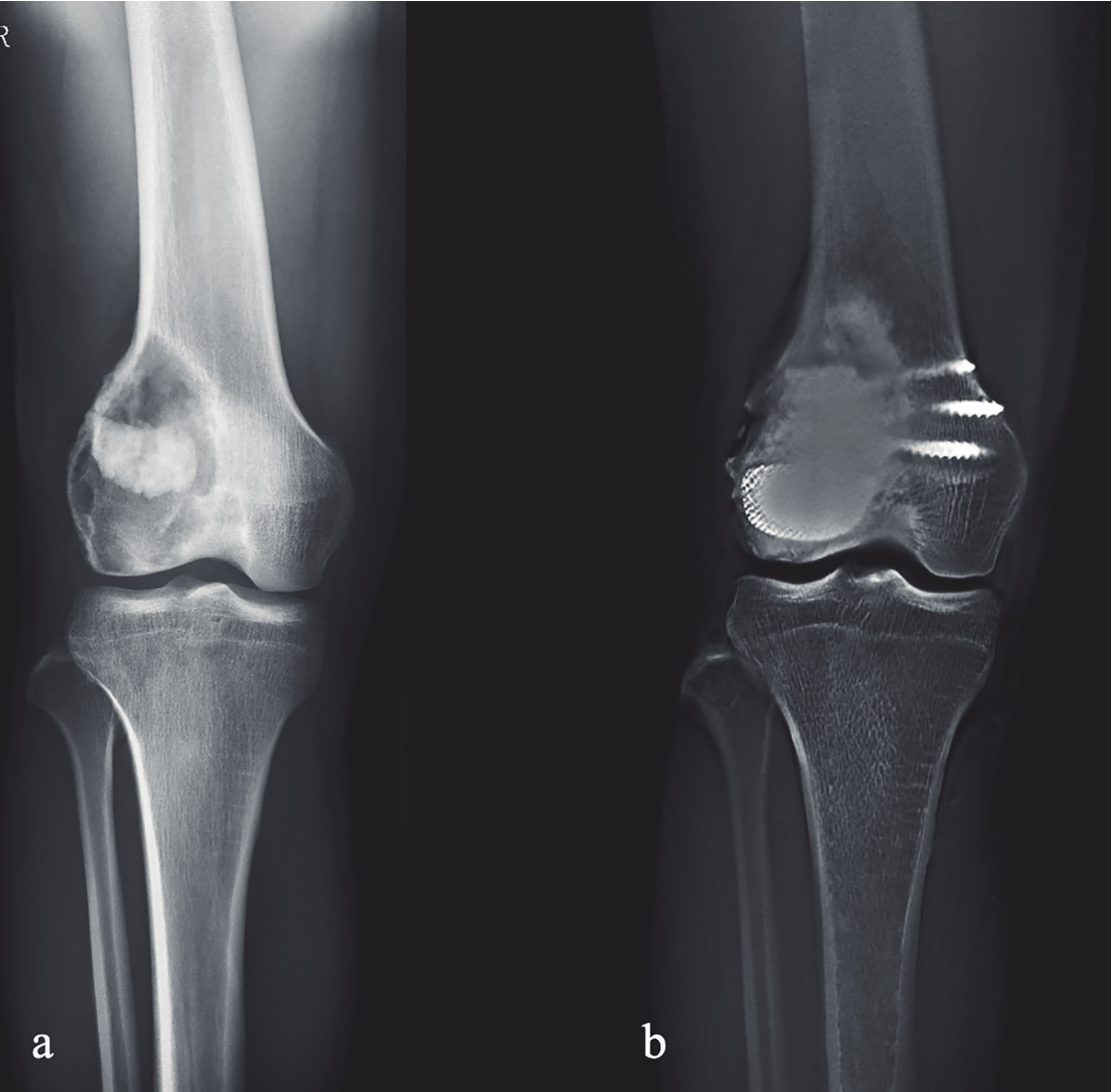
Preoperative and postoperative X-ray evaluations: **(A)** Preoperative AP view of one patient from the cohort. **(B)** Extended curettage, subchondral bone grafting and 3D-printed strut-type prosthetic reconstruction were performed; T-SMART taken at 24 months after surgery showed that the subchondral bone thickness was increased and osseointegration was achieved.

**Figure 4:**
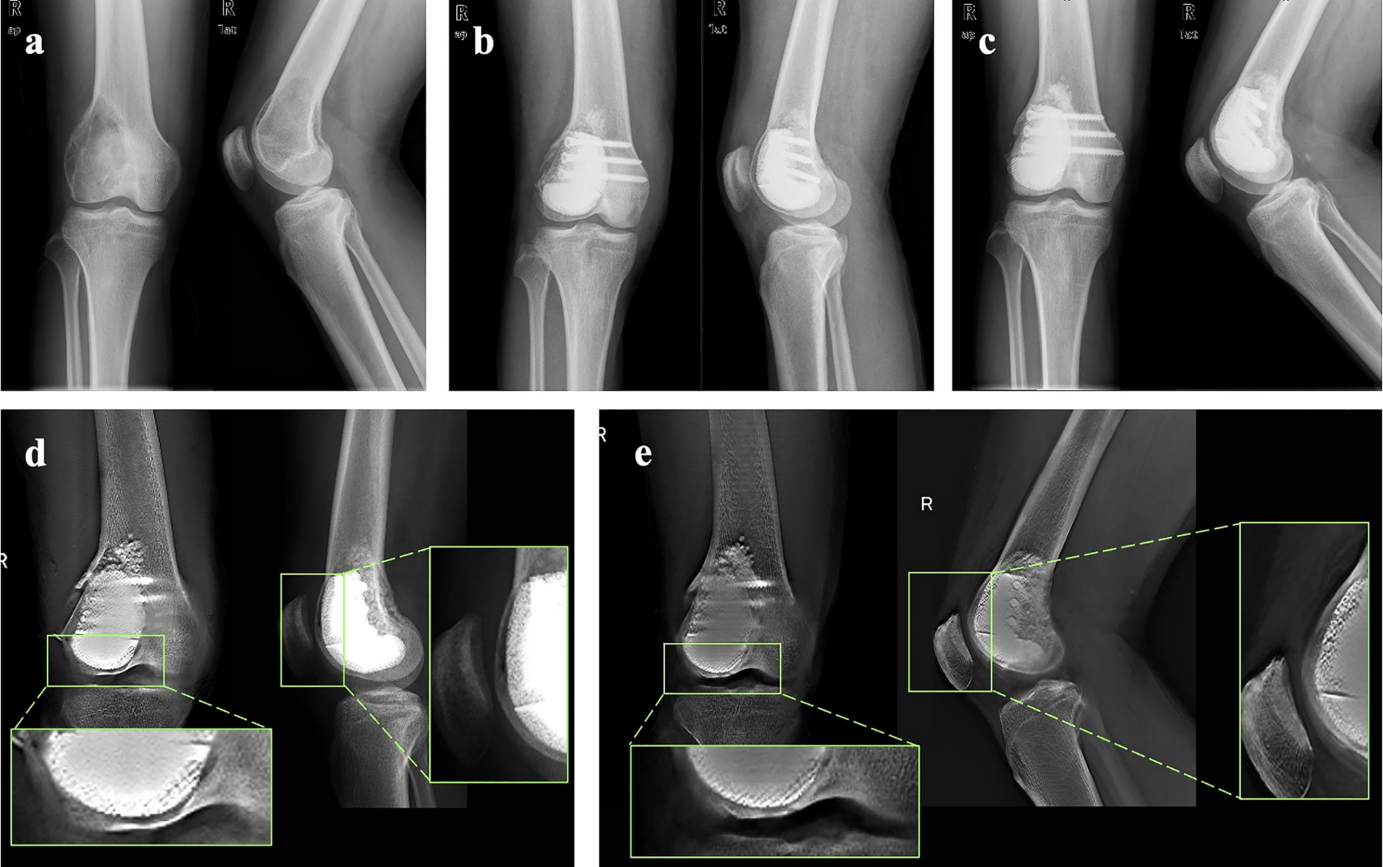
Representative case: **(A)** Preoperative X-ray of the knee of one patient from the cohort. **(B)** X-ray of the knee taken at 2 days after surgery. **(C)** X-ray of the knee taken at 24 months after surgery. **(D)** T-SMART taken at 1 day after surgery showed the interfacial gap between bone and implant (green box). **(E)** T-SMART taken at 24 months after surgery showed that the interfacial gap had disappeared (green box), indicating that excellent osseointegration was achieved.

**Figure 5:**
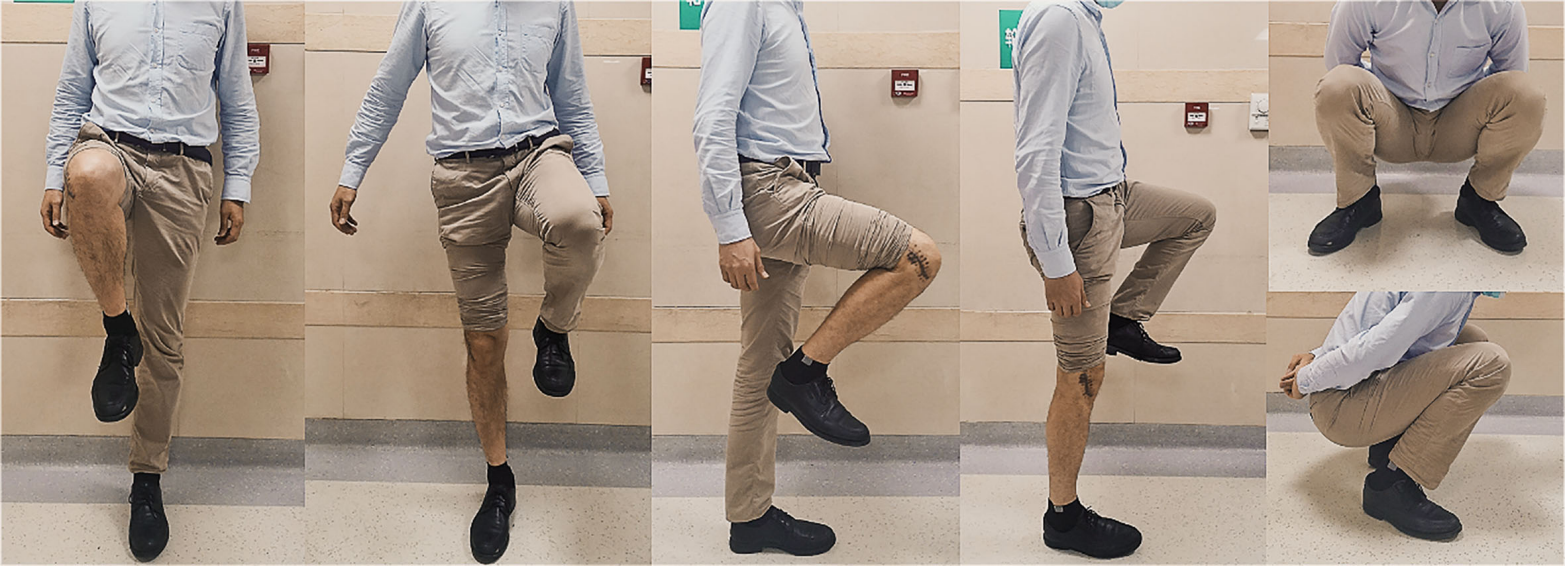
Two years after the operation, the knee function of the patient was favorable.

The baseline information of patients is presented in **Table 1**. This study was performed in accordance with the 1964 Helsinki Declaration and was authorized by the Ethics Committee of West China Hospital (approval number 2018347). All people provided written informed consent to participate in this investigation. The human research participants provided informed consent for the publication of the images in **Figures 2**–**5**.

**Table 1:** Demographics of 15 GCTB patients treated with 3D-printed strut-type prostheses.

Patients	Age (years)	Gender	Grade ^a^	Follow-up (months)	The preoperative SCB condition	
					The affected SCB percentage (%)	The residual SCB thickness (mm)
1	31	F	II	40	36.8	1.2
2	27	F	II	36	50.2	1.5
3	25	F	I	42	32.4	2.3
4	44	M	II	28	27.2	2.1
5	29	M	II	31	40.0	4.0
6	29	M	II	28	24.1	1.7
7	35	F	II	28	25.5	2.0
8	33	F	I	26	18.2	2.6
9	24	M	II	18	32.2	2.0

a According to the Campanacci grading system.

SCB, subchondral bone.

### 2.2 Prosthesis design and fabrication

**Figure 6:**
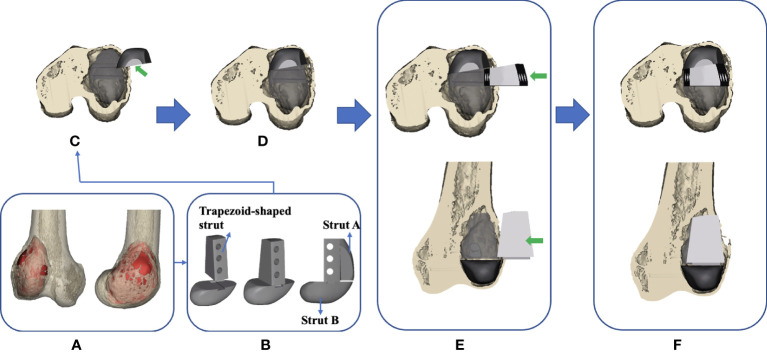
Flow chart of the design and implantation of the 3D-printed strut-type prosthesis to repair the cavity bone defect following extended curettage of GCTBs in the distal femur: **(A)** 3D models of the femur and tumor (red). **(B)** Based on the shape and size of the tumor, a modular strut-type prosthesis was created. It consisted of three components: Trapezoid-shaped strut, Strut A, and Strut B. **(C, D)** Implanting Strut A into the void to support the patellofemoral joint. **(E, F)** Implanting the Sturt B into the void to achieve axial support and maintain the stability of the tibiofemoral joint.

All prostheses were customized for each patient by our clinical team and fabricated (Chunli Co, Ltd., Tongzhou, Beijing, China) with an electron beam melting technique (ARCAM Q10plus; Mol̈ndal, Sweden). Firstly, the preoperative femoral CT data were used to build virtual 3D femur models in Mimics V16.0 software (Materialise Corp., Leuven, Belgium); the size and shape of cavity bone defects caused by tumorous destruction had also been evaluated. Afterward, the prosthesis prototype was created by Geomagic Studio software (Geomagic Inc., Morrisville, United States) using the initial data (**Figure 6A**). After removing unnecessary features, smoothing the surface of the prosthesis, and dividing the prosthesis into solid structural and porous structural regions, the definitive prosthesis with a modular system which can perfectly match the tumor-induced defect has been created (**Figure 6B**).

The modular system design facilitates the minimization of the size of cortical windows and conservation of bone stock and makes it convenient for assembling the components in a limited space. The design concept of “strut-type prosthesis” and additional details related to the modular system had been described thoroughly in our previous biomechanical study (34) (**Figure 6C–F**).

3D digital preoperative planning and a surgical simulation were performed before the surgery, and the size and shape of the prosthesis might be further adjusted to ensure satisfactory fitting in the tumorous void. Finally, the data of the definitive prosthesis were imported into an electron beam melting system to manufacture the implant (**Figure 2A–D**).

### 2.3 Surgical techniques

All patients underwent surgeries performed by the same senior surgeon (Chongqi Tu). After the induction of general anesthesia, the patients were placed in a lateral position. The lateral approach of the knee joint to the affected side was selected. Main surgical procedures were performed as follows. (Step 1) A cortical fenestration was made with a mini drill and osteotome; the size of the cortical window was determined based on preoperative planning. (Step 2) After the tumor was mechanically curettage with different-sized curettes and high-speed drills, adjunct therapies including alcohol and argon beam were used to extend the tumor kill zone several millimeters. (Step 3) Autograft harvested from the iliac crest was filled under the subchondral bone area with a thickness of at least 10 mm. (Step 4) A plastic implant trial that was fabricated by stereo lithography appearance technique (UnionTech Lite 450HD, Shanghai, China) was used to confirm satisfied fit before the definitive endoprosthesis was implanted. The margin of the bony window might be trimmed to the proper size and shape to match the prosthesis, thus achieving tight contact between bone and prosthesis. The strut-type prosthesis was then implanted on the graft-bed site. (Step 5) Three screws penetrated the contralateral cortex for further fixation. A standard prosthetic radiograph was obtained immediately after the implantation using a C-arm X-ray machine to evaluate the accuracy of impanation. (Step 6) Finally, the gaps between the host bone and the prosthesis were filled with the autografted and artificial bone to form a tight biological fixation between the host bone and the prosthesis surface and achieve an integrated reconstruction (**Figure 2E**).

### 2.4 Postoperative management and follow-up

After surgery, the affected limb was immobilized in a physiologically neutral position for one week. The active isometric quadriceps exercises were administered one week postoperatively, and the flexion and extension of the knee joint were allowed two weeks postoperatively. The gradual weight-bearing stance with walking aids started approximately four to six weeks postoperatively, followed by full weight-bearing gait exercise. It’s also important to note that patients did not utilize Denosumab postoperatively.

All patients underwent physical examinations (MSTS-93, and ROM), radiographs, and Tomosynthesis Shimadzu Metal Artefact Reduction Technology (T-SMAR) monthly for the first three months postoperatively, then every three months thereafter. At each follow-up visit, the subchondral bone thickness would be recorded according to the previously described method; the bone graft healing and the osteointegration of the bone/prosthesis interface were evaluated by T-SMART (**Figure 3**).

### 2.5 Statistical analysis

Data analyses were undertaken with IBM SPSS Statistics software, version 25 (IBM SPSS, Armonk, NY, USA). The normality of the continuous data was tested by Kolmogorov–Smirnov test. The MSTS-93 scores were compared using Mann–Whitney U tests; the paired-designed data including the subchondral bone thickness and the ROM were compared using paired t-test. A *P*-value <0.05 was considered statistically significant.

## 3 Results

### 3.1 Oncological outcomes and complications

All patients were followed up for 30.8 ± 7.5 months (18–42 months) after surgery, and no patient was lost to follow-up. At the final follow-up, all the patients were alive without local tumor recurrence or distant metastasis in the lung. Neither surgical-related complications, such as neurovascular injuries, wound infection, and deep venous thrombus (DVT), nor prosthesis-related complications, such as aseptic loosening, breakage, periprosthetic fracture, and periprosthetic infection, were observed.

### 3.2 Bone graft healing and implant osseointegration

All autografts exhibited osseous union at the graft-host junction with a mean time of 3.3 ± 0.4 months (range, 3.0-4.0 months). Furthermore, excellent osseointegration of the bone/prosthesis interface was observed on the T-SMART at a mean time of 4.1 ± 0.6 months (range, 3.5-5.0 months). The representative cases of autograft healing and implant osseointegration are presented in **Figure 4**.

### 3.3 Articular cartilage and subchondral bone thickness

The baseline of the area of affected subchondral bone (AASB) and subchondral bone thickness before surgery was 31.8% ± 9.6% (range, 18.2% ~50.2%) and 2.2 ± 0.8 mm (range, 1.2-4.0 mm), respectively. As determined by the Kolmogorov–Smirnov test, the preoperative and postoperative subchondral bone thickness and the ROM all obeyed normal distribution (*P* > 0.05). At the last follow-up, the mean subchondral bone thickness increased to 10.9 ± 1.3 mm (range, 9.1-12.1 mm). This difference was statistically significant (p < 0.05, paired t-test).

In addition, no degenerative changes in the knee joint, such as the collapse of the articular surface and articular degeneration, were found. A summary of the follow-up is shown in **Table 2**.

**Table 2:** Follow-up and outcome assessment.

Patients	Preoperative data		Graft bone union time (months)	Implant osseointegration time (months)	Last follow-up data			Complications
	MSTS	ROM			MSTS	ROM	The residual SCB thickness (mm)	
1	17	0-115	3.0	4.0	26	0-120	11.5	NONE
2	15	0-105	4.0	4.5	24	0-115	9.3	NONE
3	22	0-110	3.0	3.0	27	0-135	12.1	NONE
4	21	0-110	3.5	4.5	30	0-130	11.2	NONE
5	24	0-115	3.0	4.0	30	0-135	11.5	NONE
6	20	0-105	3.5	4.5	28	0-125	9.1	NONE
7	12	0-100	3.0	5.0	24	0-115	13.0	NONE
8	20	0-115	4.0	4.0	27	0-120	10.5	NONE
9	18	0-100	3.0	3.5	24	0-110	9.8	NONE

SCB, subchondral bone.

### 3.4 Knee function

Patients had significant improvements in postoperative MSTS-93 score and ROM compared with their preoperative statuses (post-operative vs. pre-operative): MSTS-93 score: (26.7 ± 2.4) vs. (18.8 ± 3.7), *P* < 0.05, Mann–Whitney *U* tests; ROM: (108.3° ± 6.1°) vs. (122.8° ± 9.1°), *P <* 0.05, paired *t*-test. (**Figure 5**)

## 4 Discussion

GCTB occurs most frequently in the distal femur where the tumor can result in osteolytic destruction and invasion of the subchondral bone and the articular cartilage. The subchondral bone in conjunction with the articular cartilage is considered an osteochondral unit, allowing for shock absorption, the elasticity of compression, and knee joint stability (35). Maintaining the articular subchondral bone thickness and preventing further damage to it holds great importance in the treatment of GCTB in the distal femur. However, the current mainstream treatment method, namely extended intralesional curettage followed by cement packing combined with subchondral bone grafting, is still deficient in protecting the articular subchondral bone (36). To address this problem, in this study we designed a 3D-printed strut-type prosthesis with a porous titanium surface. Following extended intralesional curettage, this novel prosthesis was used for the reconstruction of cavity bone defects. The primary objectives of our approach were to enhance osseointegration, protect the articular surface, and mitigate the risk of osteoarthritis.

We found that the articular subchondral bone was well protected and satisfied clinical and radiographic outcomes had been achieved in virtually all patients after a mean follow-up period of 30.8 ± 7.5 months. Compared with the preoperative measurements, these patients were associated with substantial increases in subchondral bone thickness (p < 0.05, paired t-test). Radiographic findings at the last follow-up showed that the mean subchondral bone thickness was 10.9 ± 1.3 mm (range, 9.1-12.1 mm), which represents a nearly four-fold increase compared with that before surgery. Additionally, improved ROM (p < 0.05, paired t-test) and knee functional score (*P* < 0.05, Mann–Whitney *U* tests) have also been observed while no degeneration changes of the knee joint were found. These finds extend to those of Chen et al. (15), suggesting that a higher quantity of subchondral bone remaining can reduce the risk of postoperative mechanical failure and degeneration of the articular surface. Mohamed et al. (14), also reported that when the subchondral bone thickness was less than 10mm, the incidence of degenerative changes in the knee joint was more than 2.5 times greater than that when the subchondral bone thickness was more than 10 mm. Similar results have been reported by Teng et al. (16) who reported that the subchondral bone layer less than 3.3 mm indicated a higher risk of mechanical failure postoperatively. Then, an important question here is: how does this reconstruction method with a newly designed prosthesis contribute to the protection of the subchondral bone after extended intralesional curettage and the promotion of the subchondral bone ingrowth? The main causes include the following three aspects.

Firstly, the titanium-based prosthetic reconstruction avoided the thermal damage of the traditional cementation-based reconstruction. Even though the thermal polymerization during the cement hardening process is thought to cause tumor necrosis and reduce the risk of local recurrence, this situation is analogous to the “tumoricidal effect of chemotherapeutic drugs”, which can affect both healthy and cancerous cells. The tumoricidal ability of cementation is at the expense of delayed/non‐union of the bone graft and a high risk of articular subchondral bone damage. However, some may concern that the lack of tumoricidal ability by thermal polymerization of bone cement would lead to poor prognosis due to recurrence. After extended intralesional curettage supplemented by a series of mechanical and/or chemical adjuvant therapies, including the use of high-speed burr, ethanol, and argon beam, the tumor was completely removed, and the postoperative local recurrence rate can already be maintained at a low-level (37, 38). Therefore, considering the shortcomings of using bone cement here, the benefits of non-cementation reconstruction overweigh the harms associated with a lack of tumoricidal ability.

Secondly, the porous titanium surface with specific size and porosity provided excellent biocompatibility. The elastic modulus of the solid titanium is significantly higher than human bone tissue. However, after introducing a porous structure, the stiffness of the material can be lowered by almost an order of magnitude, which would be favorable for osteoconductivity and osseointegration. According to the study of Torres-Sanchez et al. (39), the porous scaffold with a pore size of 500 um and 70% porosity can simulate the trabecular bone. In our study, the 3D-printed strut-type prostheses with porous structures were designed and fabricated based on these parameters. By providing a similar elastic modulus to the graft bone, the use of this prosthesis not only helps to avoid the stress imbalance often seen when PMMA bone cement is used due to its dissimilar elastic modulus with the host bone, but also reduces the risk of further damage to the subchondral grafting layer. Such stress imbalance could contribute to the destruction of the articular surface, leading ultimately to osteoarthritis. Our previous study constructed a 3D finite element solid model of a distal femoral bone defect, which was reconstructed using the 3D-printed strut-type prosthesis, to study femur biomechanics in a representative daily activity. The Finite Element (FE) results revealed a near-normal stress distribution in both the femur post-curettage and the 3D-printed strut-type prosthetic reconstruction, as well as in the prosthesis itself. This confirms that this reconstruction method is biomechanically reliable (34). Furthermore, unlike bone cement, our porous titanium prosthesis closely mimics the biological compatibility and bioactivity of bone, fostering an environment conducive to bone integration, a key aspect lacking in PMMA bone cement-based reconstructions.

Thirdly, it is worth mentioning the increasing attention towards CPC as an alternative in bone cementation (40). Despite its biocompatibility advantages over PMMA, and a lower cost and potential infection rate when compared to 3D-printed prostheses, CPC is not without shortcomings (26). The material possesses lower mechanical strength than PMMA, potentially contributing to early construct failure under weight-bearing conditions (41). Additionally, the unpredictable resorption rate of CPC, which may outpace new bone formation and leave voids, presents a potential risk (42). The porous titanium surface of the prosthesis, thanks to its excellent osseointegration capability, promoted peri-prosthesis bone ingrowth. In this study, the initial interfacial gap between the implant and the graft/host bone in the T-SMART gradually disappeared over time, indicating that osseointegration between the bone and implant has been achieved. Osseointegration describes a structural and functional complex that occurs on the prosthesis/bone interface which is critical for the longevity and durability of a prosthesis after implantation into the body (43). The remodeling of the graft into the host bone and the full bony ingrowth into the porous surface resulted in an implant-graft-host bone complex, which prevented the micromotion. By contrast, since the osseointegration of the cement surface is unachievable and there is a significant difference in elastic modulus between the cement and graft bone, the surrounding bone would be expected to be gradually absorbed under long-term stress, resulting in the “sclerotic rim” (9). Some believed that the sclerotic rim might allow micromotion between the cement and graft bone, leading to fretting wear of the articular subchondral bone and poor bone graft incorporation (44). Taken together, in contrast with the traditional cementation reconstruction, the 3D-printed strut-type prosthetic reconstruction has several advantages, including non-thermal reconstruction, excellent biocompatibility, and osteointegration capacity of the porous titanium surface. The 3D-printed strut-type prosthesis combined with autograft reconstruction can achieve the effective reconstruction of the cavity bone defect with the articular subchondral bone being protected.

## 5 The limitation and expectation

There are some limitations in this study. Firstly, we acknowledge that the sample size is relatively small, which may limit the generalizability of our findings. Secondly, the duration of follow-up in our study is relatively short. We are aware that the long-term prognosis of patients, especially in terms of the incidence of osteoarthritis, cannot be fully assessed based on our current follow-up period. Therefore, we will continue to enroll more patients and extend the follow-up period in our ongoing studies to more comprehensively evaluate the long-term performance and impact of the newly designed prosthesis. Thirdly, the main contribution of this work is that we proposed an alternative to the existing cementation reconstruction method aimed at reducing the risk of subchondral bone damage. Despite the introduction of CPC as an another alternative to PMMA, which is reported to have a lower cost than 3D-printed prosthesis and potentially lower infection rate, it has not been widely adopted. Future work should therefore include follow-up follow up studies that compare the outcomes between different surgical modalities such as cementation, CPC, and the use of the 3D-printed prosthesis. Finally, the design and fabrication of 3D-printed customized prostheses are a multi-step and time-consuming process, typically taking 1-2 weeks. Patients should be preoperatively informed of the risk of tumor progression during this waiting period. For those scenarios where immediate reconstruction is critical, CPC, with its advantages of lower cost, reduced risk of surgical complications such as infection, and bioactivity, might present a viable alternative solution.

## 6 Conclusion

3D-printed strut-type prosthesis combined with autograft reconstruction has advantages such as biocompatibility, osseointegration capacity, and subchondral bone protection. Based on our results, it provides acceptable early functional and radiographic outcomes in patients with grade I or II GCTB in the distal femur. Therefore, if our early outcomes can be further validated in studies with more patients and sufficient follow-up, this method might be a feasible and effective alternative for the treatment of Campanacci grade I or II giant cell tumor in the distal femur with the affected subchondral bone area after extended intralesional curettage.

## Data availability statement

The original contributions presented in the study are included in the article/supplementary material. Further inquiries can be directed to the corresponding authors.

## Ethics statement

Written informed consent was obtained from the individual(s) for the publication of any potentially identifiable images or data included in this article.

## Author contributions

Study conception and design: LM and CT. Performing the surgeries: CT. Analysis and interpretation of data: YZ and YG. Drafting of manuscript: LT and YL. Manuscript revision: CT, XH, ML, and LM. And all authors read the manuscript and approved the submission. All authors contributed to the article and approved the submitted version.
